# Examining the Role of Oxytocinergic Signaling and Neuroinflammatory Markers in the Therapeutic Effects of MDMA in a Rat Model for PTSD

**DOI:** 10.3390/ph17070846

**Published:** 2024-06-27

**Authors:** Haron Avgana, Roni Shira Toledano, Irit Akirav

**Affiliations:** 1 Department of Psychology, School of Psychological Sciences, University of Haifa, Haifa 3498838, Israel; haroonavgana@gmail.com (H.A.); ronitol1988@gmail.com (R.S.T.); 2 The Integrated Brain and Behavior Research Center (IBBRC), University of Haifa, Haifa 3498838, Israel

**Keywords:** MDMA, PTSD, oxytocin, fear extinction, neuroinflammation

## Abstract

MDMA-assisted psychotherapy has shown potential as an effective treatment for post-traumatic stress disorder (PTSD). Preclinical studies involving rodents have demonstrated that MDMA can facilitate the extinction of fear memories. It has been noted that MDMA impacts oxytocin neurons and pro-inflammatory cytokines. Thus, the aim of this study was to explore the role of oxytocinergic signaling and neuroinflammatory markers in the therapeutic effects of MDMA. To achieve this, male rats were subjected to a model of PTSD involving exposure to shock and situational reminders. MDMA was microinjected into the medial prefrontal cortex (mPFC) before extinction training, followed by behavioral tests assessing activity levels, anxiety, and social function. Our findings indicate that MDMA treatment facilitated fear extinction and mitigated the shock-induced increase in freezing, as well as deficits in social behavior. Shock exposure led to altered expression of the gene coding for OXT-R and neuroinflammation in the mPFC and basolateral amygdala (BLA), which were restored by MDMA treatment. Importantly, the OXT-R antagonist L-368,899 prevented MDMA’s therapeutic effects on extinction and freezing behavior. In conclusion, MDMA’s therapeutic effects in the PTSD model are associated with alterations in OXT-R expression and neuroinflammation, and MDMA’s effects on extinction and anxiety may be mediated by oxytocinergic signaling.

## 1. Introduction

Post-traumatic stress disorder (PTSD) is a multifaceted neuropsychiatric condition that may develop after experiencing a traumatic event, as defined by the fifth edition of the Diagnostic and Statistical Manual for Mental Disorders (DSM-V). Recently, 3,4-methylenedioxymethamphetamine (MDMA)-assisted psychotherapy gained clinical attention for treating treatment-resistant PTSD [1,2,3].

MDMA shares pharmacological similarities to amphetamines and classical psychedelics [4]. However, it elicits a distinct psychoactive experience, leading some to classify it as an “entactogen,” derived from Latin: touching within [5]. Among its positive effects, MDMA can induce euphoria, reduce anxiety, and enhance empathy [6].

In animal models, MDMA has been observed to facilitate the extinction of fear memory and modulate fear memory reconsolidation and social behavior [7,8,9,10,11,12]. However, the underlying mechanisms of MDMA’s therapeutic effects are not well understood. MDMA has a complex polypharmacological effect, acting as a potent monoamine agonist stimulating the efflux of serotonin (5-HT), dopamine, and norepinephrine [13,14]. Additionally, MDMA robustly facilitates the secretion of the neuropeptide oxytocin in both the peripheral and central nervous systems (CNS) [15,16,17].

Oxytocin has gained attention as a potential treatment for PTSD because of its involvement in processes disrupted in PTSD, such as fear extinction, stress regulation, and pro-sociability [18,19,20,21]. Another mechanism underlying MDMA’s therapeutic action in PTSD may involve its immunomodulatory effects [22]. PTSD is characterized by elevated levels of pro-inflammatory cytokines, such as interleukin-1β (IL-1β), tumor necrosis factor-α (TNF-α), and IL-6, both in humans and in rodent models [22,23,24,25,26,27,28]. Additionally, accumulating evidence suggests that oxytocin can attenuate CNS inflammation [19,29,30].

### Top of Form

Therefore, the present study investigated the involvement of oxytocin- and neuroinflammatory markers in mediating MDMA’s therapeutic-like effects in a rat model of PTSD. In this study, we focused on the medial prefrontal cortex (mPFC) and the basolateral amygdala (BLA), as these regions have been extensively implicated in fear memory and extinction. In Experiment 1, we examined whether micro-injection of MDMA into the mPFC enhances fear extinction and its effects on mRNA expression of the oxytocin receptor (OXT-R) and neuroinflammatory markers in both the mPFC and the BLA. In Experiment 2, we assessed whether blocking oxytocin signaling in the mPFC through micro-injection of a selective OXT-R antagonist would block MDMA’s therapeutic-like effects.

## 2. Results

### 2.1. The Impact of MDMA on Behavior in Rats Subjected to Shock and Reminders

**Figure 1:**
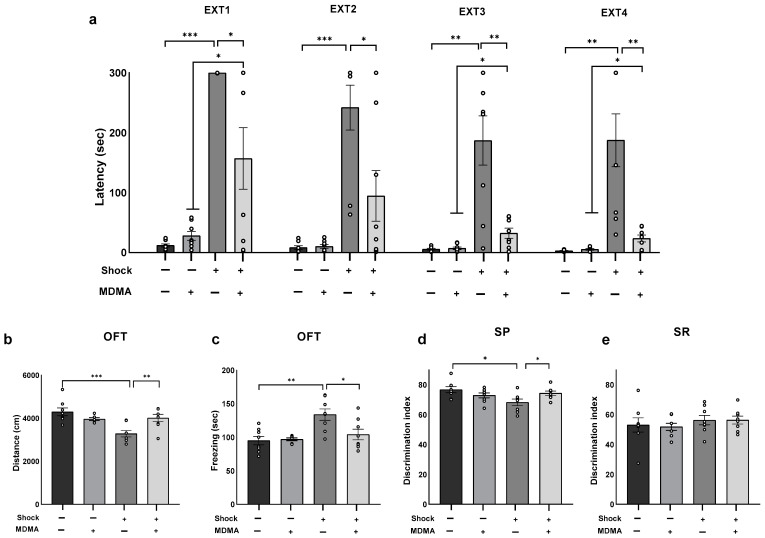
The impact of MDMA on behavior in rats subjected to shock and reminders. In comparison to the No Shock–Veh and the Shock–MDMA groups, the Shock–Veh group exhibited (**a**) increased latency to enter the dark compartment on Ext1–Ext4, (**b**) decreased distance travelled in the OFT, (**c**) increased freezing levels in the OFT, and (**d**) decreased discrimination index in the social preference test, (**e**) with no difference in discrimination index in the social recognition test. Ext: extinction; OFT: open field test; SP: social preference; SR: social recognition. *, *p* < 0.05, **, *p* < 0.01, ***, *p* < 0.001. *n* = 8 for all groups.

**Scheme 1:**
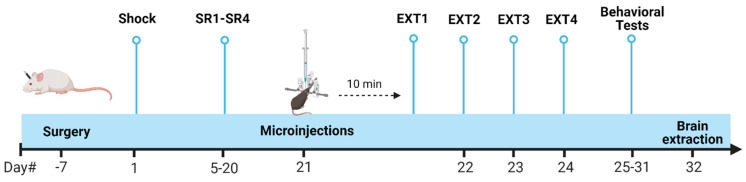
A diagram illustrating the experimental design.

For extinction (Figure 1a), repeated-measures ANOVA with main factors of shock, drug, and extinction day (2 × 2 × 4) was used (see Scheme 1 for experimental design). Significant effects of drug [F(1,28) = 19.513, *p* < 0.001], shock [F(1,28) = 79.135, *p* < 0.001], day [F(3,84) = 9.603, *p* < 0.001], shock × drug [F(1,28) = 22.546, *p* < 0.001), and shock × day [F(3,84) = 6.001, *p* < 0.001) interactions were found. Post-hoc analysis revealed that the Shock–Veh group demonstrated increased latency to enter the dark chamber in comparison with the No Shock–Veh group on Ext 1,2 (*p* < 0.001) and Ext 3,4 (*p* < 0.01). This indicates that exposure to shock and reminders impaired extinction. The Shock–Veh group demonstrated increased latency compared to the Shock–MDMA group on Ext 1,2 (*p* < 0.05) and Ext 3,4 (*p* < 0.01) groups, suggesting that MDMA facilitated extinction in rats exposed to shock and reminders. In addition, the Shock–MDMA group demonstrated increased latency compared to the No Shock–MDMA group (Ext 1,3,4: *p* < 0.05). Last, no significant differences in latency were observed between the No Shock–Veh and No Shock–MDMA groups on any of the extinction days, suggesting that MDMA had no effect on the latency in the absence of the shock.

For distance traveled in the open field test (OFT) (Figure 1b), two-way ANOVA [shock × drug (2 × 2] indicated significant effects of shock [F(1,31) = 10.411, *p* < 0.01] and shock × drug interaction [F(2,31) = 12.819, *p* < 0.01], with no effect of drug (*p* = 0.196). An independent sample t-test revealed that the Shock–Veh group traveled less compared to the Shock–MDMA (*p* < 0.01) and the No Shock–Veh (*p* < 0.001) groups, suggesting that MDMA prevented shock-induced hypo-locomotion.

For freezing levels in the OFT (Figure 1c), two-way ANOVA indicated significant effects of shock [F(1,31) = 11.886, *p* < 0.01], drug [F(1,31) = 4.345, *p* < 0.05], and shock × drug interactions [F(2,31) = 5.638, *p* < 0.05). An independent sample t-test revealed that the Shock–Veh group exhibited increased freezing compared to the Shock–MDMA (*p* < 0.05) and the No Shock–Veh (*p* < 0.01) groups, suggesting that MDMA prevented the shock-induced elevation in anxiety-like behavior.

For social preference (SP; Figure 1d), two-way ANOVA indicated significant shock × drug interaction [F(2,31) = 7.261, *p* < 0.05], with no significant effects of shock (*p* = 0.068) or drug (*p* = 0.543). An independent sample t-test revealed that the Shock–Veh group exhibited a decreased discrimination index compared to the Shock–MDMA (*p* < 0.05) and No Shock–Veh (*p* < 0.05) groups, suggesting that MDMA prevented the shock-induced reduction in social preference.

For social recognition (SR; Figure 1e), two-way ANOVA indicated no significant main effect for shock (*p* = 0.259), drug (*p* = 0.858), or shock × drug interaction (*p* = 0.854), suggesting that MDMA did not impair social memory. The total time of exploration in the social tests was measured (Supplementary Material; Figure S1).

### 2.2. The Effects of MDMA on mRNA Levels of Oxytocin Receptor, IL-1β, IL-6, and TNF-α in Rats Exposed to Shock and Reminders

#### 2.2.1. Oxytocin Receptor (OXT-R)

**Figure 2:**
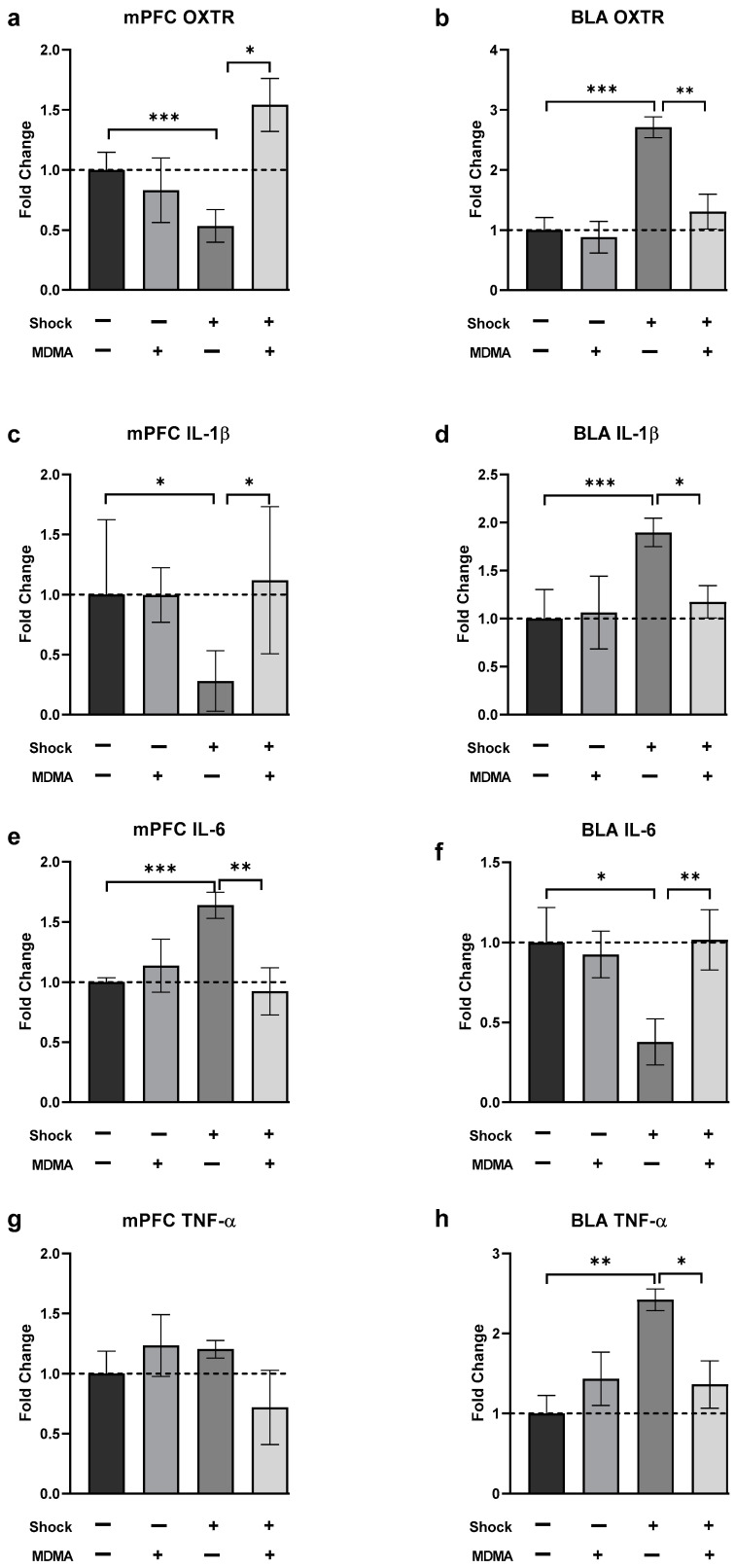
The effects of MDMA on mRNA expression of OXT-R, IL-1β, IL-6, and TNF-α in the mPFC and BLA in rats subjected to shock and reminders. In comparison to the No Shock–Veh and Shock–MDMA groups, the Shock–Veh group exhibited (**a**) decreased OXT-R expression in the mPFC, (**b**) increased OXT-R expression in the BLA, (**c**) decreased IL-1β expression in the mPFC, (**d**) increased IL-1β expression in the BLA, (**e**) increased IL-6 expression in the mPFC, and (**f**) decreased IL-6 expression in the BLA, (**g**) with no significant differences in the expression of TNF-α in the mPFC (**h**) and increased TNF-α expression in the BLA in the Shock–Veh group. *, *p* < 0.05, **, *p* < 0.01, ***, *p* < 0.001. *n* = 5–8.

In the mPFC (Figure 2a), two-way ANOVA indicated significant effects of shock [F(1,26) = 13.416, *p* < 0.01) and shock × drug interaction [F(1,26) = 5.021, *p* < 0.05), with no effect of drug [F(1,26) = 0.799, *p* = 0.379]. An independent sample t-test showed a significant reduction in OXT-R mRNA in the Shock–Veh group in comparison to the Shock–MDMA (*p* < 0.05) and No Shock–Veh (*p* < 0.001) groups, suggesting that MDMA counteracted the shock-induced reduction in mPFC OXT-R levels.

In the BLA (Figure 2b), two-way ANOVA indicated significant effects of shock [F(1,26) = 11.983, *p* < 0.01], drug [F(1,26) = 8.237, *p* < 0.01), and shock × drug interaction [F(1,26) = 4.617, *p* < 0.05]. An independent sample t-test showed a significant increase in OXT-R mRNA in the Shock–Veh group compared to the Shock–MDMA (*p* < 0.01) and No Shock–Veh (*p* < 0.001) groups, suggesting that MDMA counteracted the shock-induced elevation in BLA OXT-R.

#### 2.2.2. IL-1β

In the mPFC (Figure 2c), two-way ANOVA indicated significant effects of drug [F(1,25) = 4.766, *p* < 0.05] and shock × drug interaction [F(1,25) = 4.819, *p* < 0.05], with no effect of shock (*p* = 0.082). An independent sample t-test showed a significant reduction in mPFC-1β mRNA in the Shock–Veh group compared to the Shock–MDMA (*p* < 0.05) and No Shock–Veh (*p* < 0.05) groups, suggesting that MDMA prevented the shock-induced reduction in IL-1β.

In the BLA (Figure 2d), two-way ANOVA indicated significant effects of shock [F(1,21) = 11.773, *p* < 0.01] and shock × drug interaction [F(1,21) = 6.280, *p* < 0.05], with no effect of the drug (*p* = 0.068). An independent sample t-test showed a significant increase in IL-1β mRNA in the Shock–Veh group in comparison to the Shock–MDMA (*p* < 0.05) and No Shock–Veh (*p* < 0.001) groups, suggesting that MDMA prevented the shock-induced elevation in IL-1β.

#### 2.2.3. IL-6

In the mPFC (Figure 2e), two-way ANOVA indicated a significant shock × drug interaction [F(1,20) = 8.479, *p* < 0.05], with no significant effects of shock or drug (*p* = 0.251 and *p* = 0.084 respectively). An independent sample t-test showed a significant increase in IL-6 mRNA expression in the Shock–Veh group compared to the Shock–MDMA (*p* < 0.01) and No Shock–Veh (*p* < 0.001) groups, suggesting that MDMA prevented the shock-induced elevation in mPFC IL-6.

In the BLA (Figure 2f), two-way ANOVA indicated significant effects of shock [F(1,20) = 11.193, *p* < 0.01]) and shock × drug interaction [F(1,20) = 11.025, *p* < 0.01], with no effect of the drug (*p* = 0.424). An independent sample t-test showed a significant reduction in IL-6 mRNA expression in the Shock–Veh group compared to the Shock–MDMA (*p* < 0.01) and No Shock–Veh (*p* < 0.05) groups, suggesting that MDMA prevented the shock-induced reduction in BLA IL-6.

#### 2.2.4. TNF-α

In the mPFC (Figure 2g), two-way ANOVA indicated a significant shock × drug interaction [F(1,24) = 5.437, *p* < 0.05], with no effect of shock [F(1,24) = 1.312 *p* = 0.266] or drug [F(1,24) = 0.971 *p* = 0.336]. An independent sample t-test did not show significant differences between the Shock–Veh and the Shock–MDMA (*p* = 0.061) and No Shock–Veh (*p* = 0.081) groups.

In the BLA (Figure 2h), two-way ANOVA indicated significant effects of shock [F(1,20) = 5.404, *p* < 0.05] and shock × drug interaction [F(1,20) = 6.807, *p* < 0.05], with no effect of the drug (*p* = 0.561). An independent sample t-test showed a significant elevation in TNF-α mRNA expression in the Shock–Veh group in comparison to the Shock–MDMA (*p* < 0.05) and No Shock–Veh (*p* < 0.01) groups, suggesting that MDMA prevented the shock-induced elevation in TNF-α in the BLA.

### 2.3. Correlations between mRNA Expression and Behavior

Pearson bivariate correlations were conducted between behavior and the expression of mRNA (OXT-R, Table 1; IL-1β, IL-6, TNF-α, Table 2) in the mPFC and BLA to investigate the association between anxiety-like phenotype and the expression of oxytocin and neuroinflammation markers.

For OXT-R, in the mPFC, significant negative correlations were found with the latency in Ext 1 (r = −0.612, *p* < 0.001), Ext 2 (r = −0.599, *p* < 0.001), and Ext 4 (r = −0.612, *p* < 0.01), suggesting that decreased mPFC OXT-R levels are associated with increased latency to enter the dark compartment (i.e., enhanced fear retrieval (EXT1) and impaired extinction (EXT2, EXT4)).

In the BLA, significant positive correlations were found between OXT-R mRNA levels and latency in Ext 1 (r = 0.501, *p* < 0.01), Ext 2 (r = 0.508, *p* < 0.01), Ext 3 (r = 0.511, *p* < 0.01), and Ext 4 (r = 0.421, *p* < 0.05), suggesting that increased BLA OXT-R is associated with enhanced fear retrieval (EXT1) and impaired extinction (EXT2–EXT4).

For IL-1β in the mPFC, a significant negative correlation was found with the latency in Ext 1 (r = −0.407, *p* < 0.05), suggesting that decreased IL-1β is associated with increased fear retrieval.

In the BLA, a significant positive correlation was found with the latency on Ext 1 (r = 0.536, *p* < 0.05) and Ext 3 (r = 0.523, *p* < 0.05), suggesting that increased BLA IL-1β is associated with elevated fear retrieval and impaired extinction.

For TNF-α in the BLA, a significant positive correlation was found with the latency on Ext 1 (r = 0.645, *p* < 0.01), suggesting that elevated BLA TNF-α is associated with enhanced fear retrieval. In addition, a significant negative correlation was found between TNF-α and the social preference discrimination index (r = −0.541, *p* < 0.05), suggesting that increased BLA TNF-α is associated with reduced social preference.

### 2.4. The Effects of Co-Administration of MDMA and Oxytocin Receptor Antagonist on Behavior

We found that MDMA restored shock-induced downregulation of OXT-R in the mPFC and that the most robust correlations with the behavioral phenotype were with the expression of OXT-R. For this reason, we pharmacologically investigated whether MDMA’s effects on behavior in shocked rats are mediated through the activation of OXT-R. To that end, shocked animals were microinjected with the OXT-R antagonist L-368,899 (250 ng/0.5 µL) into the mPFC, which was immediately followed by MDMA microinjection. Other shocked groups were microinjected with MDMA, L-368,899, or vehicle alone for comparison.

**Figure 3:**
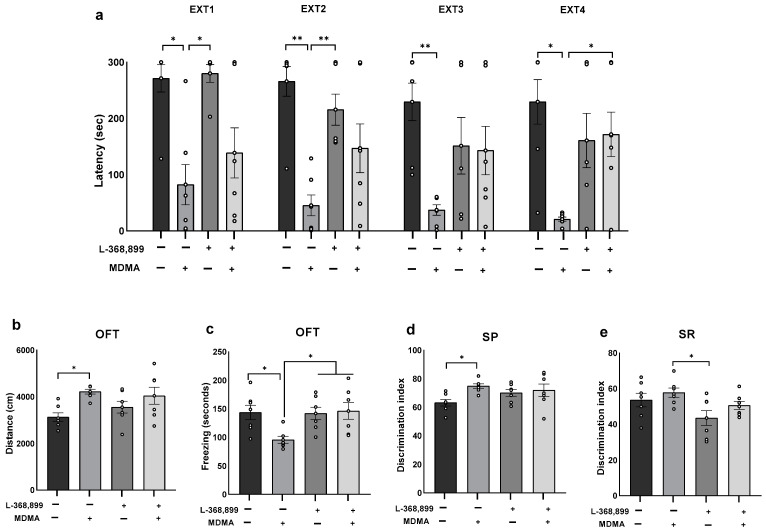
The effects of the oxytocin receptor antagonist L-368,899 on the behavior of shocked rats. (**a**) Extinction: The Shock–MDMA group exhibited decreased latency to enter the dark compartment compared to the Shock–Veh group on Ext1–Ext4 and compared to the Shock–L-368,899 group on Ext1–Ext2. Importantly, the Shock–L-368,899–MDMA group exhibited increased latency to enter the dark compartment on Ext 4 compared to the Shock–MDMA group. (**b**) In the OFT, in comparison to the Shock–Veh group, the Shock–MDMA group showed increased distance travelled (**c**) and decreased freezing. Importantly, the Shock–MDMA group showed decreased freezing compared to the Shock–L-368,899 and Shock–L-368,899–MDMA groups. (**d**) In social preference, the Shock–MDMA group exhibited increased social preference compared to the Shock–Veh group. (**e**) In social recognition, the Shock–MDMA group showed increased discrimination compared to the Shock–L-368,899 group. Ext: extinction; OFT: an open field test. *, *p* < 0.05, **, *p* < 0.01, *n* = 7.

For extinction (Figure 3a), repeated measures ANOVA [drug × day (4 × 4)] indicated significant effects of drug [F(3,24) = 8.804, *p* < 0.001] and day [F(3, 69) = 3.173, *p* < 0.05], without a significant drug × day interaction (*p* = 0.301). Post-hoc analysis showed that the Shock–Veh group exhibited increased latency in comparison to the Shock–MDMA group on all extinction days (Ext 1,4 *p* < 0.05; Ext 2,3, *p* < 0.01) as seen in Figure 1. The Shock–L-368,899 group exhibited increased latency in comparison to the Shock–MDMA group on Ext 1 (*p* < 0.05) and Ext 2 (*p* < 0.01). Importantly, the Shock–L-368,899–MDMA group showed increased latency compared to the Shock–MDMA group on Ext 4 (*p* < 0.05). These results suggest that the OXT antagonist L-368,899 blocked MDMA’s long-term facilitating effects on extinction retention. The shock–MDMA–L-368,899 group did not significantly differ from any other groups on Ext1–Ext3.

For distance travelled in the OFT (Figure 3b), one-way ANOVA indicated a significant effect of drug [F(3,27) = 4.104, *p* < 0.05)]. Post-hoc analysis showed that the Shock–Veh group significantly differed from the Shock–MDMA group (*p* < 0.05). The Shock–L-368,899–MDMA group was not significantly different from any of the other groups.

For freezing levels in the OFT (Figure 3c), one-way ANOVA indicated a significant effect of the drug [F(3,27) = 4.411, *p* < 0.05)]. Post-hoc analysis showed that the Shock–MDMA group significantly differed from the Shock–Veh, Shock–L-368,899, and Shock–L-368,899–MDMA groups (all *p* < 0.05). This suggests that L-368,899 blocked MDMA’s effects on anxiety-like behavior.

For social preference (Figure 3d), one-way ANOVA indicated a significant effect of the drug [F(3,27) = 3.007, *p* < 0.05)]. Post-hoc analysis revealed that the Shock–Veh group significantly differed from the Shock–MDMA group (*p* < 0.05). The Shock–L-368,899–MDMA group was not significantly different from any of the other groups.

For social recognition (Figure 3e), one-way ANOVA indicated a significant effect of drug [F(3,27) = 3.306, *p* < 0.05)]. Post-hoc analysis revealed that the Shock–L-368,899 group exhibited decreased social recognition in comparison to the Shock–MDMA group (*p* < 0.05), suggesting that L-368,899 may impair social memory in shocked rats.

## 3. Discussion

In this study, we demonstrate that acute intra-mPFC MDMA administration can enhance fear extinction and reverse shock-induced alterations in stress-related behaviors (i.e., hypolocomotion, freezing, and social preference), as well as restore shock-induced alterations in OXT-R expression and neuroinflammation markers in the mPFC and BLA. Furthermore, through pharmacological intervention with the OXT-R antagonist L-368,899, we found that the therapeutic-like effects of MDMA on extinction and freezing are mediated by OXT-R.

The potential mechanism of fear extinction has been suggested to underlie MDMA’s therapeutic effects in clinical trials [31]. Our finding that MDMA facilitated extinction aligns with studies in rodent models [11,12] and humans [10].

The anxiolytic effects of acute MDMA on shock-induced hypoactive locomotion and freezing levels in the OFT are consistent with previous studies highlighting MDMA’s anxiolytic potential [32,33,34,35]. However, there are reports of MDMA inducing anxiogenic long-term effects when administered intraperitoneally (i.p.) [34,36,37,38]. It is interesting to note that rodent models have shown that a systemic injection of MDMA can induce brain-wide toxic levels of 5-HT, potentially leading to anxiety-like behaviors [39,40]. Conversely, micro-infusion of MDMA produces a region-specific effect with reduced neurotoxicity, which may explain the anxiolytic effects observed in our current study [40,41].

The pro-social effect of MDMA on the shock-induced social deficits in the social preference test aligns with previous studies demonstrating MDMA’s prosocial effects [42,43]. Recently, an oxytocin-based mechanism in which MDMA re-opens a critical window for social reward and learning has been proposed [9]. This is supported by findings that pre-treatment with an OXT-R antagonist partially blocked MDMA’s prosocial effects [44].

Exposure to shock differentially affected the expression of OXT-R mRNA expression in the mPFC and BLA; however, MDMA treatment restored these divergent effects. The downregulation of OXT-R mRNA in the mPFC is consistent with previous findings indicating that exposure to different stressors, such as single prolonged stress and early life stress, decreases the expression of OXT-R mRNA, accompanied by reduced OXT-R protein expression [45,46]. The shock-induced upregulation in the BLA is in line with the observation that prenatal stress can increase OXT-R mRNA expression in the amygdala [47], but see also [45].

Changes in OXT-R expression may indicate altered ligand availability, as elevated oxytocin levels may result in downregulation and desensitization [48,49]. Consequently, continuous oxytocin infusion may reduce binding capabilities, and synthetic oxytocin treatment may mitigate stress-induced OXT-R overexpression [47,50].

A decrease in OXT-R mRNA levels in the mPFC may indicate diminished oxytocin signaling, as studies have shown that targeted downregulation of OXT-R in the mPFC led to reduced oxytocin signaling and was correlated with anxiety-like behavior [51]. Significantly, the reduction in oxytocin levels led to decreased excitation in the mPFC, which is crucial for fear extinction retention and retrieval [51,52,53]. Supporting this notion, intra-mPFC infusion of oxytocin has been shown to alleviate anxiety-like behaviors and reduce BLA activation, as indicated by c-Fos expression [54]. Therefore, a decrease in excitatory signaling from the mPFC to the BLA may lead to heightened BLA activation, a phenomenon associated with increased anxiety levels and impaired fear extinction [55,56].

MDMA restored the differential shock-induced alterations in the neuroinflammatory markers in both the mPFC and the BLA. A recent study showed that a single dose of MDMA attenuated IL-1β in the dentate gyrus of a rat PTSD model [22]. However, several studies suggest that a high dose of MDMA administered i.p. can potentially lead to an elevated neuroinflammatory response lasting several hours [57,58,59]. Interestingly, this neuroinflammatory response is not observed when MDMA is directly injected into the CNS [41,60].

The decrease observed in the mPFC contrasts with studies indicating that stress leads to an elevation of IL-1β [27,45]. However, others report no change [61] or delayed decrease in the mPFC but not in the BLA in IL-1β levels after a stressor [62]. Moreover, reduced IL-1β signaling has been linked to impaired contextual fear-related memories [63]. Consequently, both elevated and diminished IL-1β levels can affect memory functioning [63,64].

The increase in IL-1β in the BLA after stress aligns with previous studies showing that different stressors lead to increased microglial activity and increased IL-1β levels in the amygdala [27,65,66,67]. The upregulation of IL-6 in the mPFC following shock aligns with prior findings. For instance, the social defeat paradigm, lasting a week, resulted in increased IL-6 levels in the mPFC, although no significant change was noted in the amygdala [68,69]. Conversely, we observed a downregulation of IL-6 in the BLA. A similar effect was observed in the amygdala of mice exposed to a foot-shock model of PTSD [70]. Taken together, the findings suggest that the cytokine response to stress is differentially expressed in different brain regions.

However, the effects on neuroinflammation appear to be contingent upon the type of stressor. For instance, chronic stress led to downregulation of IL-6 in the brain stem, with no effect in the mPFC [71]. Additionally, elevated levels of IL-6 were observed in the amygdala shortly after stress, yet a downregulation was noted after a prolonged period [68]. Consequently, both increased and decreased levels of IL-6 have been associated with stress and anxiety-related behaviors [72,73].

The increase in TNF-α in the BLA is consistent with previous studies demonstrating that various stressors can upregulate TNF- α in the amygdala [35,67,74]. Furthermore, intra-amygdala microinjection of TNF-α resulted in excitotoxicity and impaired fear acquisition and extinction [75]. Additionally, chronic social defeat led to TNF-α elevation in the BLA but not in the PFC [76]; see also [74].

Pre-treatment with OXT-R antagonist prior to extinction resulted in abolishment of MDMA’s long-term facilitating effect upon extinction. This is in line with studies that demonstrate that mPFC oxytocin signaling can facilitate fear extinction [18,77]. In a recent paper, it was noted that OXT-R activation in the mPFC resulted in the depolarization of pyramidal neurons that project into the BLA [78]. Thus, oxytocin signaling may result in mPFC activation, which has been associated with enhanced fear extinction [52,53]. Another possible mechanism for facilitated fear extinction is through long-term potentiation (LTP) [79,80], as elevated oxytocin release has been found to facilitate LTP in the mPFC [81]. Nonetheless, to date, there is no evidence that MDMA has direct binding with OXT-R [82]; rather, some evidence suggests that the oxytincgeric release is mediated via 5-HT1A signalling [17,83]. Thus, these effects cannot be solely attributed to oxytocin signaling.

Similarly, OXT-R antagonist resulted in dampening MDMA’s anxiolytic effects. Central release of oxytocin has been associated with reduced anxiety [84]. Specifically, activation of OXT-R in the mPFC has been found to have an anxiolytic effect in rodents [66,85]. Moreover, activation of OXT-R in the mPFC may result in inhibition of the BLA, which has been associated with reduced anxiety [55,66,86].

Overall, our findings align with previous human studies indicating that MDMA can enhance fear extinction [10] and reduce PTSD symptoms [1,2,3]. Notably, these effects appear to be mediated by oxytocin signaling, which has several potential implications. Firstly, this suggests that oxytocin could serve as a supplementary treatment to selective serotonin reuptake inhibitors (SSRIs) in managing PTSD [86]. Secondly, given oxytocin’s role in facilitating interpersonal relationships, it is plausible that MDMA exerts its therapeutic effects, in part, by enhancing the therapeutic alliance [87]. Nonetheless, the exact role of oxytocin in MDMA-assisted psychotherapy remains to be elucidated.

Our findings suggest that the therapeutic effects of MDMA may be mediated through the attenuation of neuroinflammation [22]. Dysregulated inflammation is strongly associated with PTSD [88], and both SSRIs [89,90] and oxytocin [91,92] have demonstrated beneficial effects on neuroinflammatory processes. However, MDMA’s effects on neuroinflammation are likely dose-dependent, as high doses of MDMA can induce neuroinflammation and even neurotoxicity [93]. Therefore, further research is required to better understand the relationship between MDMA and neuroinflammation.

In summary, our study supports the notion that fear extinction may be the underlying mechanism in MDMA’s therapeutic effect. Moreover, we found that MDMA restores stress-induced divergent alterations in OXT-R mRNA expression and neuroinflammatory markers in brain regions related to fear memory. Of note, oxytocin signaling mediated these fear extinction-enhancing effects.

## 4. Materials and Methods

### 4.1. Subjects

Male Sprague Dawley rats (60 days old, approximately 225 g in weight; Envigo, Jerusalem, Israel) were housed in cages measuring 59 × 28 × 20 cm, with four rats per cage, according to their assigned treatment groups. The room temperature was maintained at 22 ± 2 °C under a12 h light/dark cycle (lights on at 7:00 a.m.). Water and laboratory food were provided ad libitum. All experimental procedures were approved by the University of Haifa Ethics and Animal Care Committee (approval number: #: UoH-IL-2201-107-4, approved on 6 January 2022), and steps were taken to minimize pain and discomfort.

### 4.2. Drugs

3,4-MDMA (hydrochloride) (1 µL/side) (Cayman Chemical, Ann Arbor, Michigan, USA) was dissolved in phosphate-buffered saline (PBS), and the oxytocin antagonist L-368,899 (0.5 µL/side) (Tocris Bioscience, Bristol, UK) was dissolved in isotonic saline. Dosages of the drugs were determined based on previous studies [11,94,95].

### 4.3. Surgical Procedure and Microinjection

Following anesthesia with a combination of 0.54 mg/kg domitor and 0.9 mg/kg ketamine, rats were positioned in a stereotactic apparatus. A stainless-steel guide cannula was unilaterally implanted targeting the mPFC (coordinates: anteroposterior, +3.2 mm; lateral, ±0.5 mm; ventral, −4.6 mm). The cannula was secured with acrylic dental cement and two skull screws. Animals were allowed one week to recover following the procedure before the commencement of experimental manipulations.

The stylus was removed from the guide cannula for microinjection, and a 28-gauge injection cannula extending 1.0 mm beyond the guide cannula tip was inserted. The injection cannula was connected to a Hamilton micro-syringe via PE20 polyethylene tubing, driven by a micro-infusion pump (CMA/100; Harvard Apparatus). Microinjections were administered unilaterally in a volume of 0.5 µL over 1 min. After injection, the cannula remained in place for an additional 1 min to minimize liquid drag along the injection tract before withdrawal.

### 4.4. Shock and Situational Reminders

The animals were introduced into an inhibitory avoidance apparatus (50 cm × 25 cm × 30 cm) consisting of two compartments of equal size: one light and one dark, with a guillotine door separating them [96].

#### 4.4.1. Shock

On day 1, each rat was initially placed in the light compartment and allowed to explore freely for 2 min. Afterward, the guillotine door separating the light and dark chambers was raised. Thirty seconds after the rat entered the dark chamber, the guillotine door closed, and the rat received an inescapable 1.5 mA foot shock lasting 10 s. Following the shock, the rat remained in the dark chamber for 60 s before being returned to its home cage. The non-shock group underwent the same procedure, with the shock mechanism deactivated.

#### 4.4.2. SRs

On days 5, 10, 15, and 20, the rats were placed in the light compartment for a duration of 1 min with the guillotine doors closed to prevent access to the shock compartment (to avoid extinction).

#### 4.4.3. Extinction

On day 21, rats underwent extinction trials daily for four consecutive days (extinction 1–extinction 4). The first extinction trial served as an index of fear retrieval. Each rat was initially placed in the light compartment with the guillotine door open. The latency, or the time it took for the rat to voluntarily move into the dark compartment, was recorded. If the rat did not cross over within 300 s, the experimenter gently guided it into the dark chamber. Subsequently, the opening between the compartments was closed, allowing the rat to freely explore the dark chamber for 180 s before being returned to its home cage.

### 4.5. Behavioral Tests

All rats underwent the behavioral procedures in the same sequential order, with tests being conducted at 24 h intervals. The experiments were conducted under dim lighting conditions (15–20 lx).

#### 4.5.1. Open Field Test

The open field test (OFT) was conducted in a darkened Plexiglas arena (45 × 45 × 43 cm^3^), with each session lasting 15 min. Data from the OFT were analyzed using the Ethovision XT9 behavioral tracking system (Noldus). Total distance was measured to assess general locomotion, and freezing levels were calculated for the initial 5 min of the test to evaluate novelty-related anxiety.

#### 4.5.2. Social Preference and Social Recognition

This task assesses sociability and short-term social recognition memory. Prior to the test, animals underwent three days of habituation to the Plexiglas arena (45 × 45 × 43 cm^3^). During the preference phase, an unfamiliar juvenile rat and a novel object were each placed in netted corrals (9 cm diameter), positioned in opposite corners of the arena (corner locations were counterbalanced between trials). Subsequently, the experimental rat was introduced into the arena for 5 min of exploration. After completion of the exploration period, the rat was returned to its home cage. Following a 30 min interval, the recognition phase commenced. During this phase, the experimental rat was provided with 5 min of exploration time alongside both the familiar juvenile rat and the novel juvenile rat. All trials were recorded using a Dericam, Indoor Pan/tilt IP camera M801W, USA), and a discrimination index was calculated. In social preference tests, the exploration time of the novel rat was divided by the total exploration time (object + rat). For social recognition tests, the exploration time of the novel rat was divided by the total exploration time (familiar rat + novel rat).

### 4.6. Real-Time (RT) PCR

Following sacrifice, brain tissues from the mPFC and BLA were collected (Supplemental Material; Figure S2). RNA extraction and qRT-PCR were performed to assess the expression levels of oxytocin receptor (OXT-R) and inflammatory markers IL-6, IL-1β, and TNF-α [97]. Briefly, for mRNA, 1000 ng of total RNA underwent cDNA synthesis using the qScript cDNA Synthesis Kit (Quanta Biosciences, Gaithersburg, MD, USA). Subsequently, Real-Time SYBR Green qRT-PCR amplification was carried out according to the manufacturer’s protocol (Quanta Biosciences, Gaithersburg, USA) on a Step One real-time PCR system (Applied Biosystems). Fold-change values were determined using the ddCt method relative to the housekeeping gene hypoxanthine phosphoribosyl transferase (HPRT; mRNA; brain). Primers for mRNA (Supplemental Material, Table S1) were custom-designed and synthesized by Agentek (Tel Aviv, Israel). Primer suitability was assessed based on standard curve analysis, melting curve analysis, and evaluations of linearity and threshold [98].

### 4.7. Experimental Design

On day 7, rats were implanted with cannula into the medial PFC. After a one-week recovery period, rats received a 1.5 mA foot shock for 10 s followed by exposure to contextual 1 min situational reminders (SR1-SR4) over days 5–20. Vehicle, MDMA (1 µg/0.5 µL), L-368,899 (250 ng/0.5 µL), or consecutive administration of L-368,899 and MDMA were microinjected unilaterally into the mPFC 10 min before the first extinction (EXT) trial. This was followed by three additional days of extinction trials (EXT2–EXT4) and a battery of behavioral tests, including the open field, social preference, and social recognition tests. Brain tissue extraction for RT-PCR analysis in the mPFC and BLA was conducted on day 32.

### 4.8. Statistical Analysis

The data are presented as means ± SEM. Statistical analysis included one-way ANOVA, mixed-design two-way ANOVA, repeated-measures ANOVA, independent t-tests, and Spearman bivariate correlation tests, as appropriate. Post-hoc comparisons were conducted using Tukey’s range test for equal variances and Dunnett’s T3 for unequal variances [99]. Statistical significance was set at *p* < 0.05. Data were analyzed using SPSS 27 (IBM, Chicago, IL, USA) and GraphPad 8.0.2 (Prsim, Boston, MA, USA).

## Figures and Tables

**Table 1:** Pearson correlation coefficients between OXT-R expression and behavioral assessments in rats subjected to shock and treated with MDMA.

	mPFC	BLA
Ext 1	r = −0.612 ***	r = 0.501 **
	*p* < 0.001	*p* < 0.01
Ext 2	r = −0.599 ***	r = 0.508 **
	*p* < 0.001	*p* < 0.01
Ext 3	r = −0.265	r = 0.511 **
	*p* = 0.157	*p* < 0.01
Ext 4	r = −0.612 **	r = 0.421 *
	*p* < 0.01	*p* < 0.05
OFT–Distance	r = −0.0.024	r = 0.135
	*p* = 0.901	*p* = 0.477
OFT–Freezing	r = −0.161	r = 0.2
	*p* = 0.394	*p* = 0.288
SP-DI	r = 0.193	r = 0.269
	*p* = 0.306	*p* = 0.151
SR-DI	r = −0.188	r = 0.083
	*p* = 0.320	*p* = 0.664

mPFC: medial prefrontal cortex; BLA: basolateral amygdala; Ext: extinction; OFT: open field test; SP: social preference; DI: discrimination index; SR: social recognition; *, *p* < 0.05, **, *p* < 0.01, *** *p* < 0.001.

**Table 2:** Pearson correlation coefficients between neuroinflammatory levels and behavioral assessments in rats subjected to shock and treated with MDMA.

	mPFC			BLA		
	IL-1β	IL-6	TNF-α	IL-1β	IL-6	TNF-α
Ext 1	r = −0.407 *	r = 0.121	r = 0.066	r = 0.536 *	r = −0.384	r = 0.645 **
	*p* < 0.05	*p* = 0.573	*p* = 0.758	*p* < 0.05	*p* = 0.105	*p* < 0.01
Ext 2	r = −0.364	r = 0.28	r = 0.148	r = 0.416	r = −0.012	r = 0.457
	*p* = 0.062	*p* = 0.184	*p* = 0.491	*p* = 0.068	*p* = 0.961	*p* = 0.056
Ext 3	r = −0.371	r = 0.280	r = 0.082	r = 0.523 *	r = −0.369	r = 0.001
	*p* = 0.057	*p* = 0.184	*p* = 0.703	*p* < 0.05	*p* = 0.120	*p* = 0.999
Ext 4	r = −0.364	r = 0.303	r = 0.037	r = 0.412	r = −0.432	r = 0.269
	*p* = 0.062	*p* = 0.149	*p* = 0.865	*p* = 0.071	*p* = 0.065	*p* = 0.280
OFT–Distance	r = −0.248	*p* = 0.075	r = 0.2	r = 0.245	r = −0.126	r = −0.225
	*p* = 0.213	*p* = 0.728	*p* = 0.349	*p* = 0.299	*p* = 0.477	*p* = 0.369
OFT–Freezing	r = −0.2	r = 0.105	r = 0.093	r = 0.270	r = −0.069	r = 0.181
	*p* = 0.316	*p* = 0.626	*p* = 0.666	*p* = 0.250	*p* = 0.780	*p* = 0.472
SP- DI	r = −0.058	r = 0.078	r = 0.083	r = 0.234	r = 0.129	r = −0.541 *
	*p* = 0.780	*p* = 0.718	*p* = 0.701	*p* = 0.320	*p* = 0.578	*p* < 0.05
SR-DI	r = −0.317	r = 0.198	r = −0.048	r = −0.090	r = −0.070	r = −0.168
	*p* = 0.114	*p* = 0.353	*p* = 0.822	*p* = 0.707	*p* = 0.764	*p* = 0.479

mPFC: medial prefrontal cortex; BLA: basolateral amygdala; Ext: extinction; OFT: open field test; SP: social preference; DI: discrimination index; SR: social recognition. *, *p* < 0.05, **, *p* < 0.01.

## Data Availability

Data are contained within the article and Supplementary Materials.

## Supplementary Materials

The following supporting information can be downloaded at: https://www.mdpi.com/article/10.3390/ph17070846/s1, Table S1. Primers for mRNAs are used for real-time PCR. Figure S1. The effects of MDMA in rats exposed to shock and reminders on exploration time in the social preference and recognition tests; Figure S2: Brain regions for molecular analysis.
