# Supplementary material for: Examining the Role of Oxytocinergic Signaling and Neuroinflammatory Markers in the Therapeutic Effects of MDMA in a Rat Model for PTSD
Source: Pharmaceuticals (Basel). 2024 Jun 27;17(7):846. doi: 10.3390/ph17070846 (PMC11279644; doi:10.3390/ph17070846)
Supplement: Supplementary file 1 [file pharmaceuticals-17-00846-s001.zip › pharmaceuticals-3053499-supplementary.pdf]

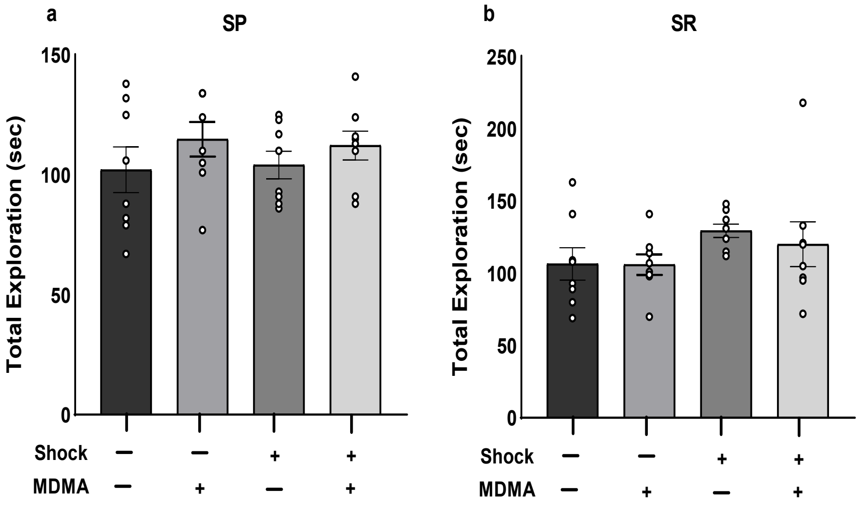

Figure S1. The effects of MDMA in rats exposed to shock and reminders on exploration time in the social preference and recognition tests. No significant differences were observed between groups in total exploration time (a) In the social preference and (b) social recognition test. SP: social preference; SR: social recognition. n = 8 for all groups.

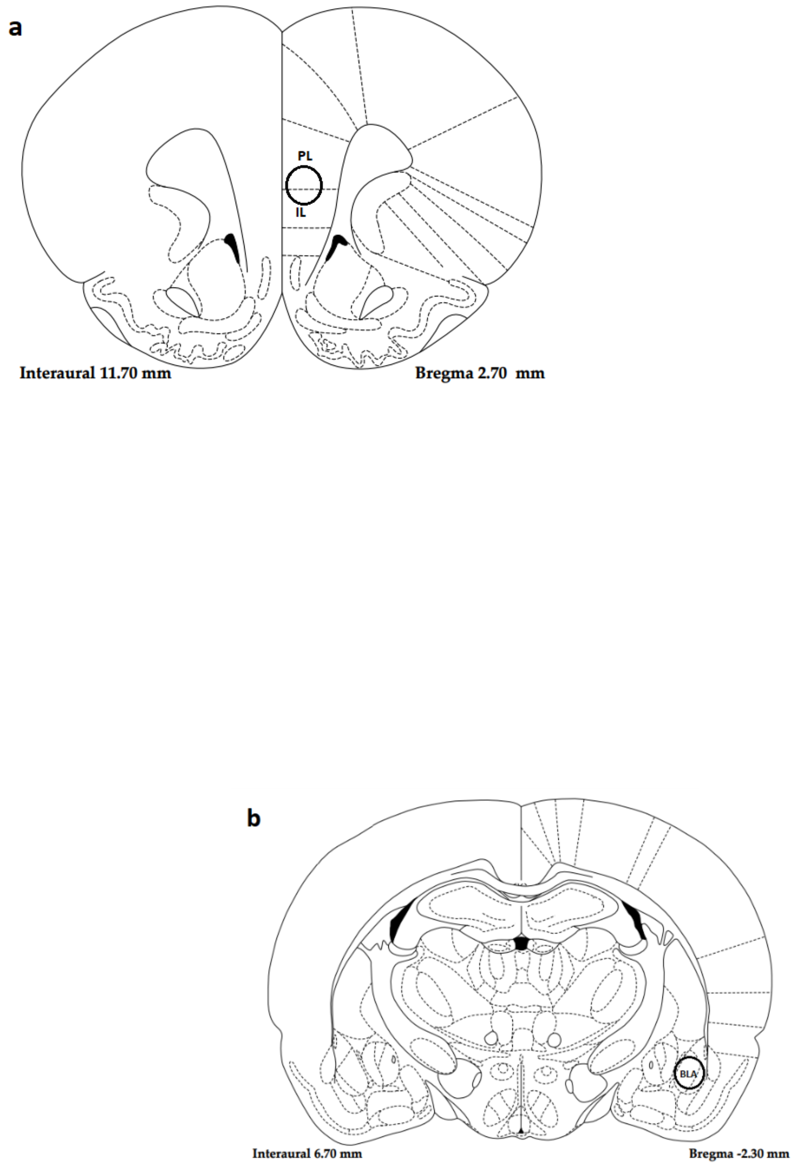

Figure S2: Brain regions for molecular analysis. Coronal view from rat brain atlas; samples were obtained with punches (1mm diameter). Numbers refer to the distance from Bregma. **(a)** IL 2.70mm anterior to bregma; **(b)** BLA -2.30mm posterior to bregma. IL: infralimbic, BLA: basolateral amygdala.

Table S1. Primers for mRNAs are used for real-time PCR.

Name	Description	Gene Bank ID (NM)	Protein Name	Primer Sequence
hprt	Housekeeping gene; used as a reference gene	NM_012583.2	HPRT	F: 5′GAGCACTTCAGGGATTTGAATCA3′<br>R: 5′GTAGATTCAACTTGCCGCTGCTGTCT3′
Oxt-r	Oxytocin receptor	NM_012871.4	OXT-R	F: 5′AATGCGCCCAAGGAAGCT3′<br>R: 5′GCACGAGTTCGTGGAAGA3′
Il1b	Interleukin 1 beta	NM_031512.2	IL-1beta	F: 5′GCTGTGGCAGCTACCTATGTCTT3′<br>R: 5′GTCACAGAGGACGGGCTCTTC3′
Il6	Interleukin 6	NM_012589.2	IL-6	F: 5′CTTCCAAACTGGATATAACCAGG3′<br>R: 5′CTTCACAAACTCCAGGTAGAAAC3′
tnf	Tumor necrosis factor alpha	NM_012675.3	TNF-alpha	F: 5′CCAGACCCTCACACTCAGATC3′<br>R: 5′CTCCGCTTGGTGGTTTGCTA3′

F: forward primer; R: reverse primer.
